# Supplementary material for: Effects of In Vitro Fermentation of Polysialic Acid and Sialic Acid on Gut Microbial Community Composition and Metabolites in Healthy Humans
Source: Foods. 2024 Feb 2;13(3):481. doi: 10.3390/foods13030481 (PMC10855092; doi:10.3390/foods13030481)
Supplement: Supplementary file 1 [file foods-13-00481-s001.zip › foods-2834788-supplementary.pdf]

Supplementary Materials

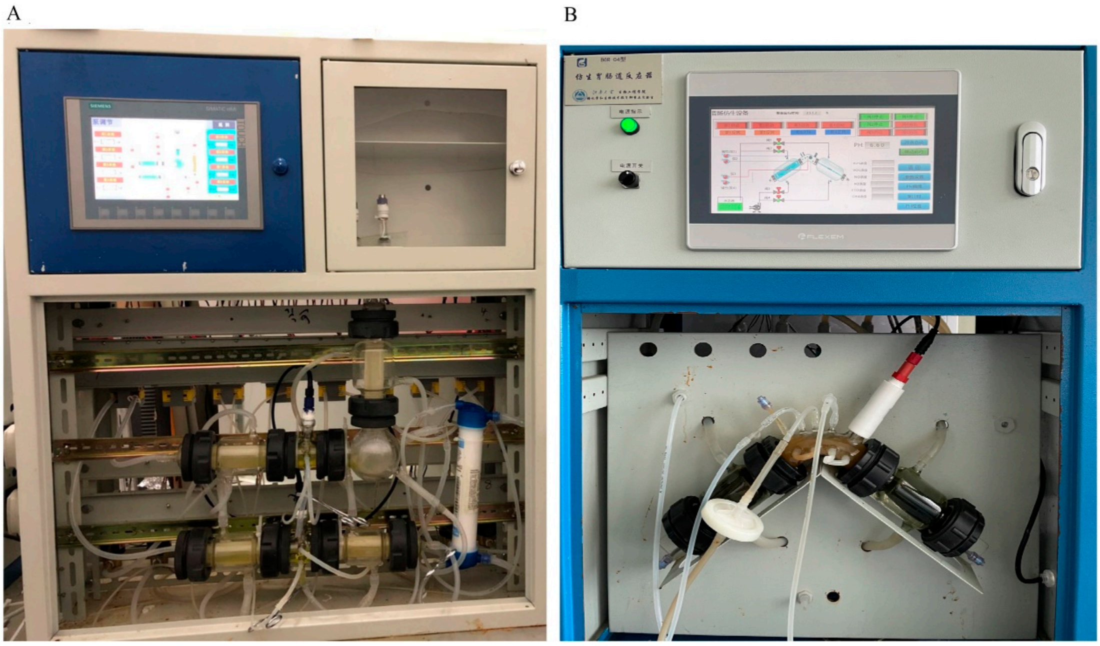

Figure S1. Structural diagram of BGR. (A) Digestive system (stomach and small intestine). (B) Fermentation system (colon). The two parts can be connected as a whole or work independently.

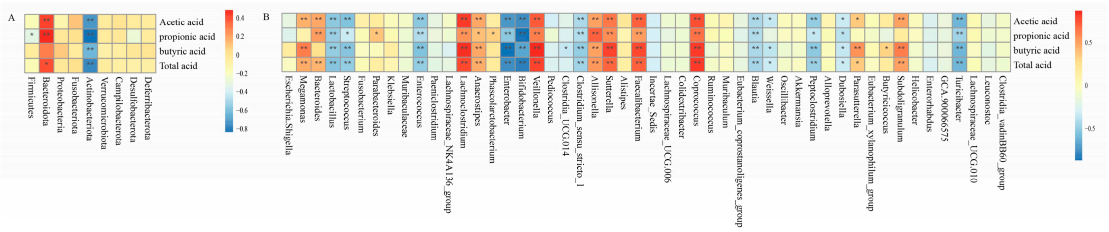

Figure S2. Spearman analysis of correlation between different species and SCFAs. (A) Correlation analysis at the phylum level. (B) Correlation analysis at the genus level.

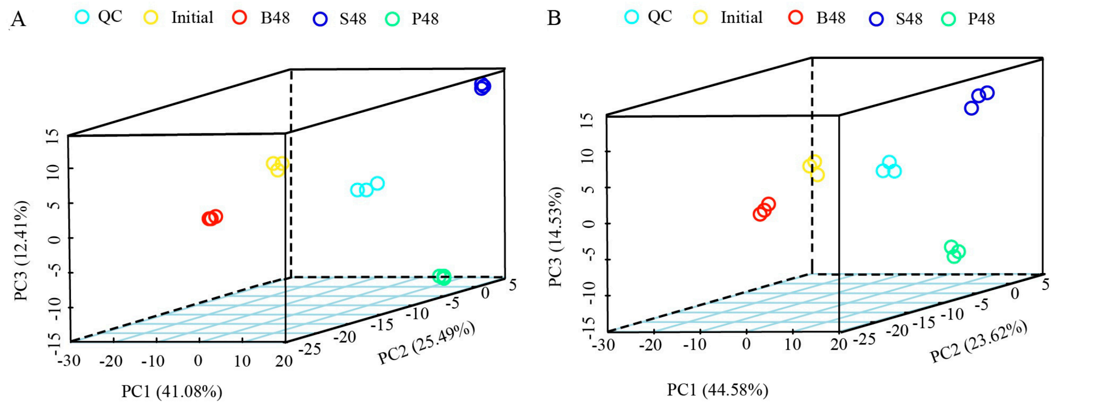

Figure S3. PCA of QC samples and samples from different experimental groups. (A) POS mode. (B) NEG mode. QC, Initial, B48, S48, and P48 indicate the QC sample, the initial point of fermentation, and the blank, SA, and PSA groups, respectively.

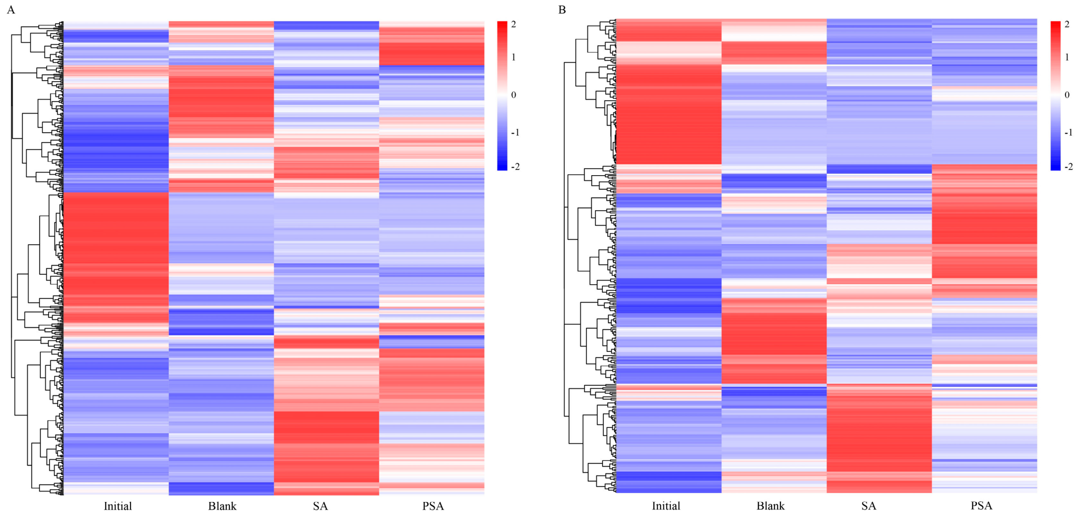

Figure S4. HCA of differential metabolites. (A) POS mode. (B) NEG mode.

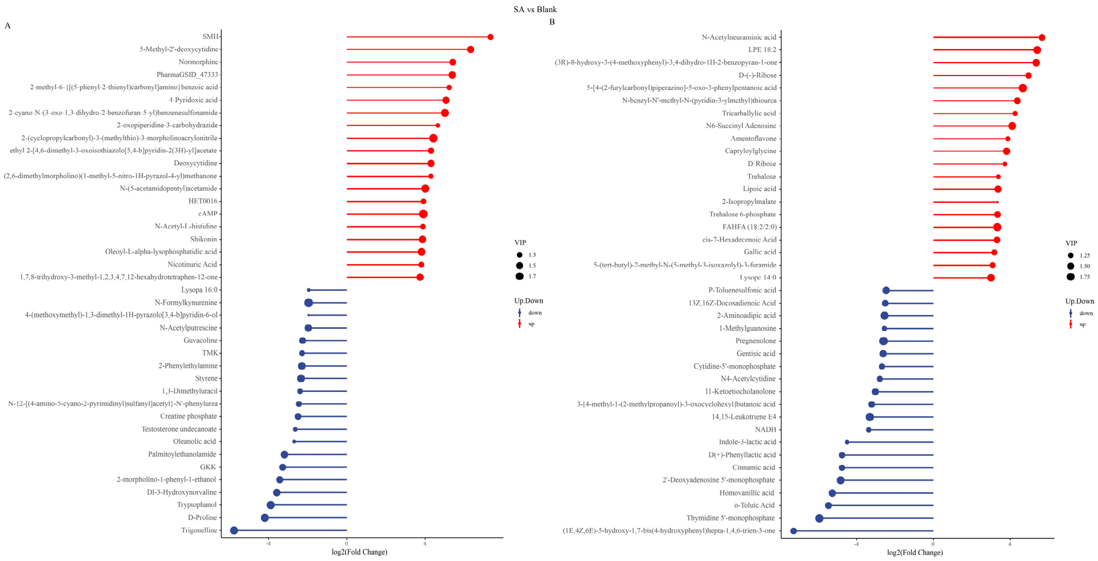

Figure S5. Matchstick charts of the top 20 differential metabolites between the SA and blank groups. (A) POS mode. (B) NEG mode. Red and blue colors indicate the up-regulated and down-regulated metabolites, respectively, and the sizes of the circles refer to different VIP values.

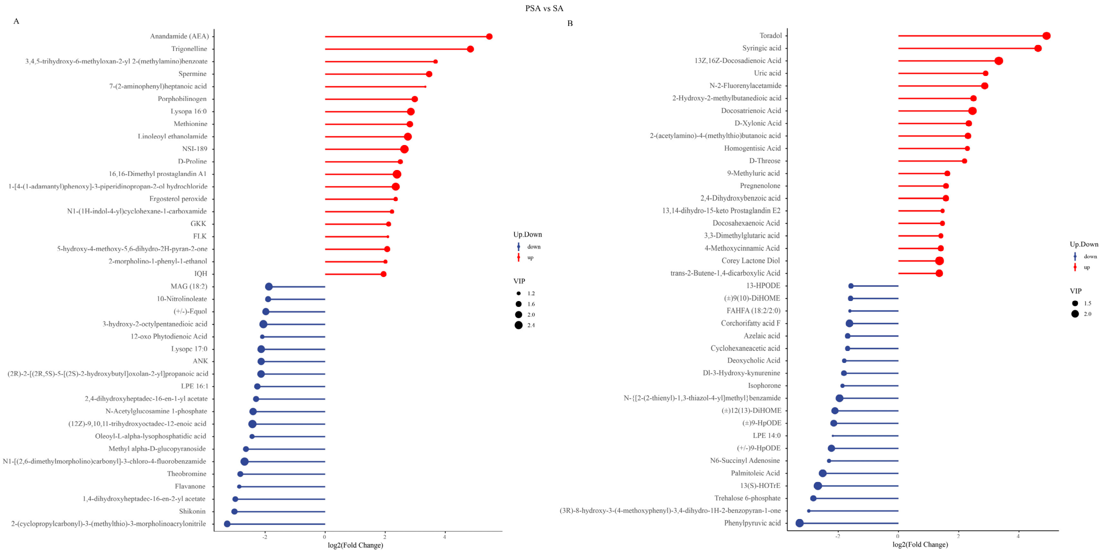

Figure S6. Matchstick charts of the top 20 differential metabolites between the PSA and SA groups. (A) POS mode. (B) NEG mode. Red and blue colors indicate the up-regulated and down-regulated metabolites, respectively, and the sizes of the circles refer to different VIP values.

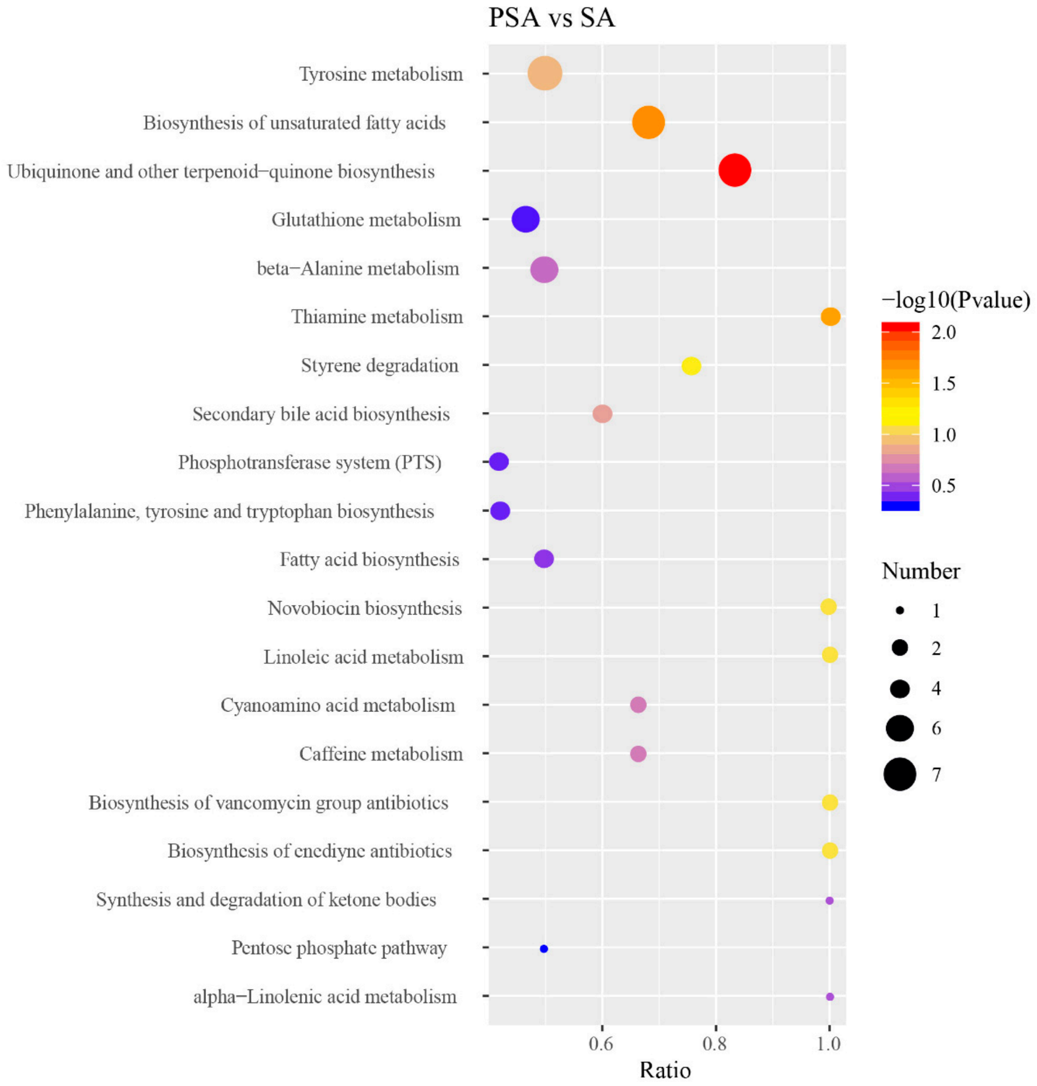

Figure S7. Metabolic pathways associated with differential metabolite changes between the PSA and SA groups. The x-coordinate is x/y (the number of differentiated metabolites in the corresponding metabolic pathway/the total number of identified metabolites in the pathway). The color of dots indicates the *P*-value of the hypergeometric test, and their size denotes the number of differential metabolites in the corresponding pathway.

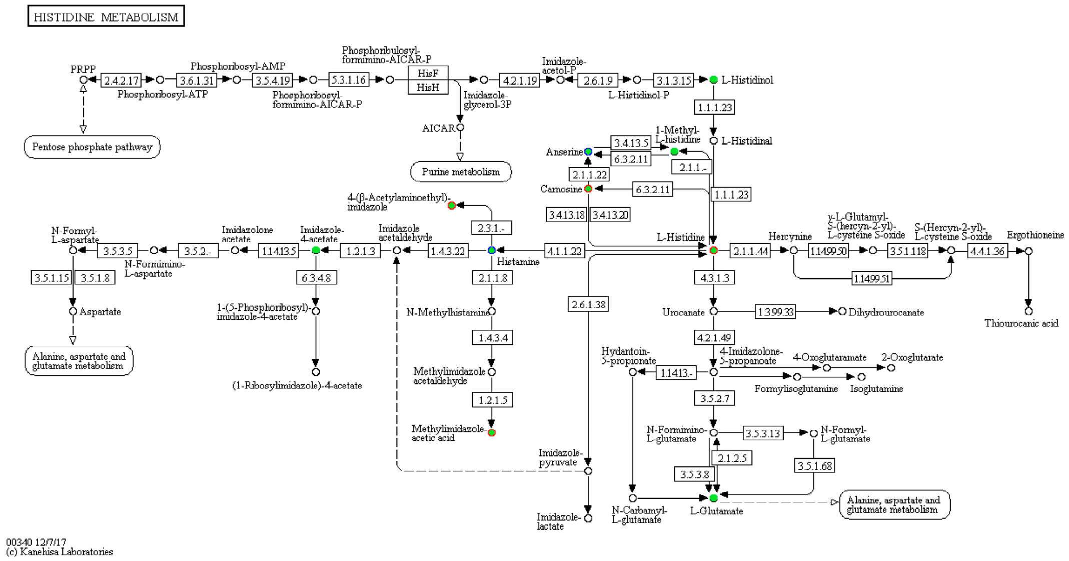

Figure S8. Major pathway maps of differential metabolite enrichment between PSA and blank. Solid green circles are noted metabolites, red circles are up-regulated differential metabolites, blue circles comprise down-regulated differential metabolites, and half circles represent metabolites that were not significantly up-regulated.

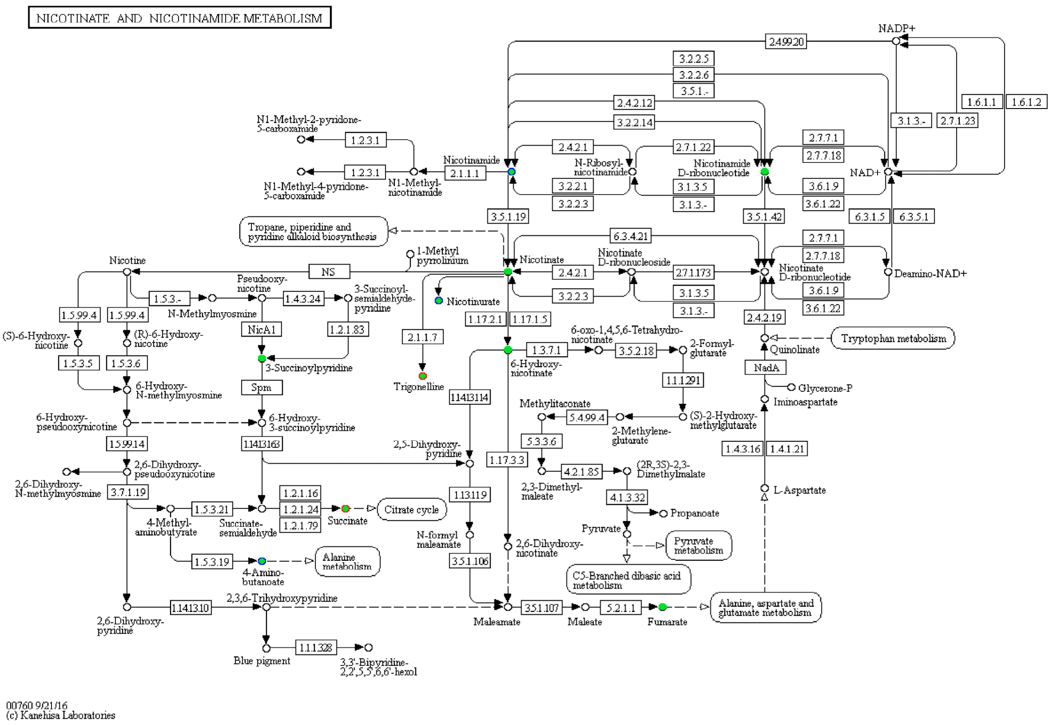

Figure S9. Major pathway maps of differential metabolite enrichment between SA and blank. Solid green circles are noted metabolites, red circles are up-regulated differential metabolites, blue circles comprise down-regulated differential metabolites, and half circles represent metabolites that were not significantly up-regulated.

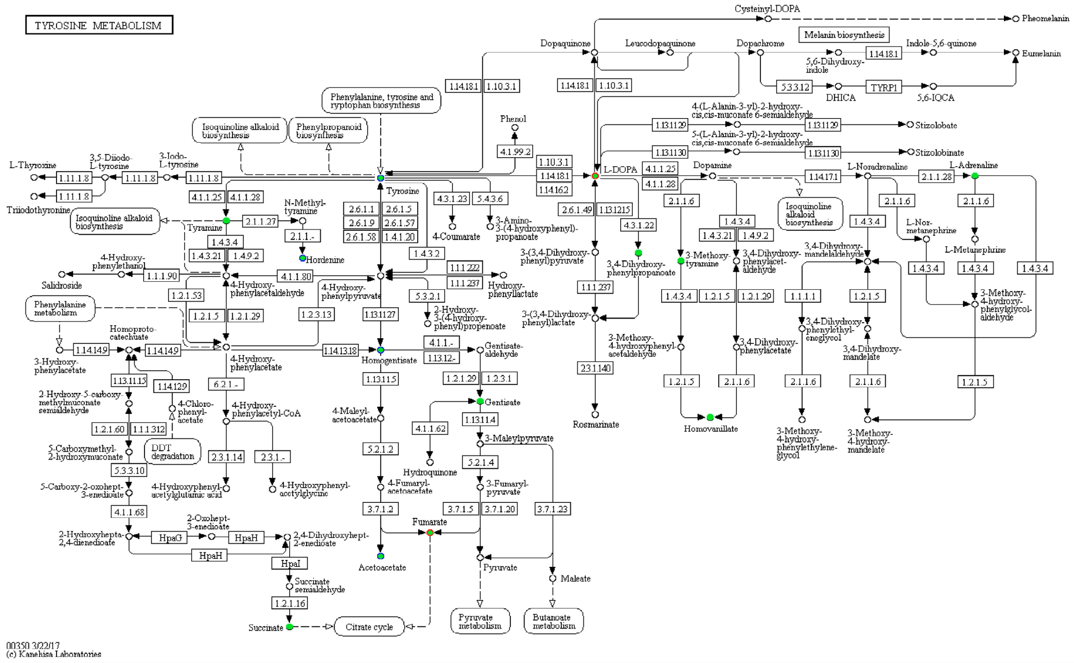

Figure S10. Major pathway maps of differential metabolite enrichment between PSA and SA. Solid green circles are noted metabolites, red circles are up-regulated differential metabolites, blue circles comprise down-regulated differential metabolites, and half circles represent metabolites that were not significantly up-regulated.

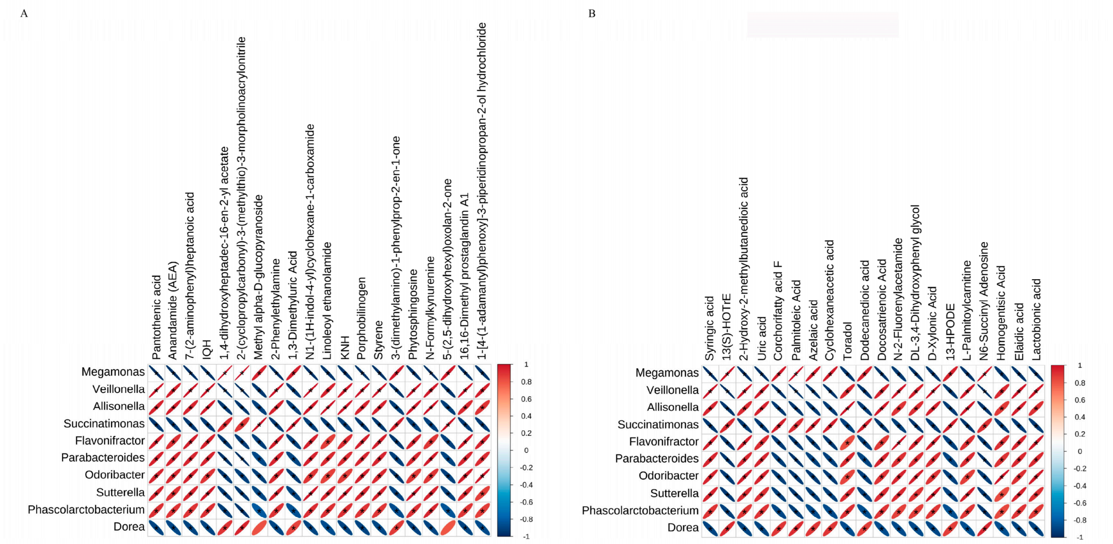

Figure S11. Correlation heat maps of different bacterial genera and metabolites between PSA and SA at the genus level. (A) POS mode. (B) NEG mode. The abscissa represents the differential metabolites, and the ordinate refers to the differential bacteria. The legends reveal the correlation coefficient, with red and blue colors indicating positive and negative correlations, respectively. The asterisk mark indicates statistical difference; $P < 0.05$.

Table S1. Consumption of PSA and SA during fecal fermentation. Different block capitals in identical columns (A, B, C, and D) indicate significant differences within the same experimental group, and different lowercase letters (a and b) on identical lines denote significant differences between groups; $P < 0.05$.

Phase	PSA (mg)	SA (mg)
0–12 h	36.4±0.17Cb	74.4±0.56Da
12–24 h	93.6±0.89Ba	90.0±1.94Ca
24–36 h	96.0±0.43Ab	104.4±1.38Aa
36–48 h	99.6±1.05Aa	99.6±0.88Ba
